# 6-(3,5-Dimethyl­benz­yl)-5-ethyl-1-[(2-phenyl­eth­oxy)meth­yl]pyrimidine-2,4(1*H*,3*H*)dione

**DOI:** 10.1107/S1600536812009841

**Published:** 2012-03-10

**Authors:** Nasser R. El-Brollosy, Mohamed I. Attia, Hazem A. Ghabbour, Suchada Chantrapromma, Hoong-Kun Fun

**Affiliations:** a Department of Pharmaceutical Chemistry, College of Pharmacy, King Saud University, PO Box 2457, Riyadh 11451, Saudi Arabia; b Crystal Materials Research Unit, Department of Chemistry, Faculty of Science, Prince of Songkla University, Hat-Yai, Songkhla 90112, Thailand; c X-ray Crystallography Unit, School of Physics, Universiti Sains Malaysia, 11800 USM, Penang, Malaysia

## Abstract

In the title pyrimidine derivative, C_24_H_28_N_2_O_3_, the uracil unit is essentially planar with an r.m.s. deviation of 0.0054 (1) Å for the eight non-H atoms. The pyrimidine ring is tilted by a dihedral angle of 77.08 (7)° with respect to the aromatic ring of the 3,5-dimethyl­benzyl substituent, whereas it is nearly parallel to the benzene ring of the pheneth­oxy­methyl unit, with a dihedral angle of 8.17 (8)°. An intra­molecular C—H⋯O hydrogen bond generates an *S*(6) ring motif. In the crystal, mol­ecules are linked by a pair of amide–uracil N—H⋯O hydrogen bonds into an inversion *R*_2_^2^(8) dimer. These dimers are stacked along the *b* axis through π–π inter­actions with a centroid–centroid distance of 3.9517 (8) Å. Weak C—H⋯π inter­actions are also present.

## Related literature

For bond-length data, see: Allen *et al.* (1987 ▶). For details of hydrogen-bond motifs, see: Bernstein *et al.* (1995 ▶). For background to anti-viral HIV therapies, see: El-Brollosy *et al.* (2007 ▶, 2008 ▶, 2009 ▶); Hopkins *et al.* (1996 ▶, 1999 ▶). For related structures, see: El-Brollosy *et al.* (2011 ▶, 2012 ▶).
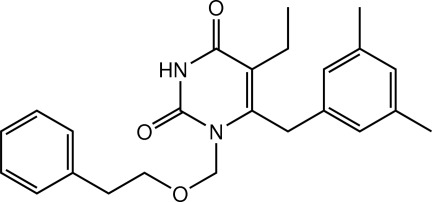


## Experimental

### 

#### Crystal data

C_24_H_28_N_2_O_3_
*M* *_r_* = 392.48
Triclinic, 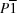

*a* = 8.34310 (1) Å
*b* = 8.47760 (1) Å
*c* = 15.9821 (2) Å
α = 91.5660 (1)°
β = 91.7820 (1)°
γ = 109.8350 (1)°
*V* = 1061.97 (1) Å^3^
*Z* = 2
Cu *K*α radiation
μ = 0.65 mm^−1^
*T* = 296 K
0.58 × 0.40 × 0.31 mm

#### Data collection

Bruker SMART APEXII CCD area-detector diffractometer
Absorption correction: multi-scan (*SADABS*; Bruker, 2009 ▶) *T*_min_ = 0.705, *T*_max_ = 0.824
13575 measured reflections
3863 independent reflections
3542 reflections with *I* > 2σ(*I*)
*R*_int_ = 0.022

#### Refinement

*R*[*F* ^2^ > 2σ(*F* ^2^)] = 0.044
*wR*(*F* ^2^) = 0.123
*S* = 1.05
3863 reflections
270 parameters
H atoms treated by a mixture of independent and constrained refinement
Δρ_max_ = 0.27 e Å^−3^
Δρ_min_ = −0.18 e Å^−3^

### 

Data collection: *APEX2* (Bruker, 2009 ▶); cell refinement: *SAINT* (Bruker, 2009 ▶); data reduction: *SAINT*; program(s) used to solve structure: *SHELXTL* (Sheldrick, 2008 ▶); program(s) used to refine structure: *SHELXTL*; molecular graphics: *SHELXTL*; software used to prepare material for publication: *SHELXTL* and *PLATON* (Spek, 2009 ▶).

## Supplementary Material

Crystal structure: contains datablock(s) global, I. DOI: 10.1107/S1600536812009841/is5086sup1.cif

Structure factors: contains datablock(s) I. DOI: 10.1107/S1600536812009841/is5086Isup2.hkl

Supplementary material file. DOI: 10.1107/S1600536812009841/is5086Isup3.cml

Additional supplementary materials:  crystallographic information; 3D view; checkCIF report

## Figures and Tables

**Table 1:** Hydrogen-bond geometry (Å, °) *Cg*3 is the centroid of the C15–C20 ring.

*D*—H⋯*A*	*D*—H	H⋯*A*	*D*⋯*A*	*D*—H⋯*A*
N2—H1*N*2⋯O1^i^	0.889 (18)	1.969 (18)	2.8558 (15)	175.7 (17)
C14—H14*B*⋯O3	0.97	2.36	3.0349 (18)	126
C21—H21*B*⋯*Cg*3^ii^	0.96	2.90	3.845 (3)	170
C22—H22*C*⋯*Cg*3^iii^	0.96	2.92	3.861 (3)	166

Symmetry codes: (i) 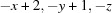; (ii) 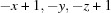; (iii) 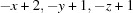.

**Fig. 1.**
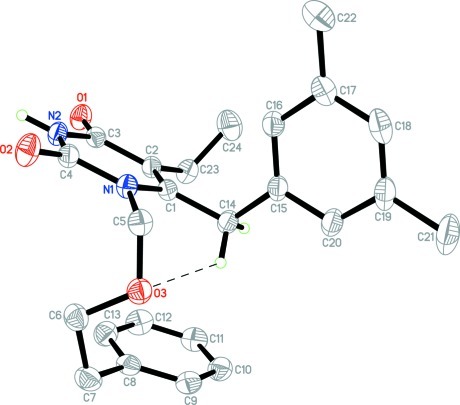
The structure of the title compound, showing 30% probability displacement ellipsoids and the atom-numbering scheme. Only the non-aromatic benzyl H and amide H atoms were shown for clarify.

**Fig. 2.**
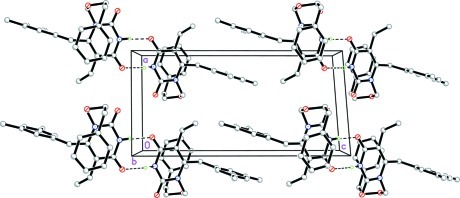
The crystal packing diagram of the title compound, viewed along the b axis. Only H atoms involving in intermolecular hydrogen bonds are drawn for clarify. N—H···O hydrogen bonds are shown as dashed lines.

## supplementary crystallographic information

### Comment

Non-nucleoside reverse transcriptase inhibitors (NNRTIs) are very promising therapies in the treatment of human immunodeficiency virus (HIV) (Hopkins *et al.*, 1996, 1999). In continuation to our interest in NNRTIs (El-Brollosy *et al.*, 2007, 2008, 2009), and as part of on-going structural studies of pyrimidine derivatives (El-Brollosy *et al.*, 2011), we synthesized the title compound (I) as a potential non-nucleoside reverse transcriptase inhibitor. It showed a good antiviral activity against HIV-1 (El-Brollosy *et al.*, 2009). Herein, the crystal structure of (I) was reported.

In the title pyrimidine derivative, C_24_H_28_N_2_O_3_, the uracil unit is essentialy planar with an r.m.s. deviation of 0.0054 (1) Å for the eight non-H atoms (C1–C4/N1/N2/O1/O2). The 3,5-dimethylbenzyl unit is also planar with an r.m.s. deviation of 0.0049 (2) Å for the nine non-H atoms (C14–C22). The pyrimidine ring is tilted with the aromatic ring (C15–C20) of the 3,5-dimethylbenzyl substituent by a dihedral angle of 77.08 (7)°, whereas nearly parallel to the C8–C13 benzene ring of the phenylethoxymethyl unit with a dihedral angle of 8.17 (7)°. The dihedral angle between the two benzene rings (C8–C13 and C15–C20) is 82.14 (8)°. The orientation of the ethyl and phenethoxymethyl groups respected to the uracil can be indicated by the torsion angles C1–C2–C23–C24 = -85.87 (19)°, and C6–O3–C5–N1 = -67.31 (14)° and C5–O3–C6–C7 = 159.11 (12)°. Intramolecular weak C14—H14B···O3 interaction generates an S(6) ring motif (Fig. 1) (Bernstein *et al.*, 1995). The bond distances in (I) are within normal ranges (Allen *et al.*, 1987) and comparable to the related structures (El-Brollosy *et al.*, 2011, 2012).

In the crystal packing, (Fig. 2), the two molecules are linked by a pair of N2—H1N2···O1 hydrogen bonds (Table 1) into a centrosymmetric *R*^2^_2_(8) dimer (Bernstein *et al.*, 1995). These dimers are stacked along the *b* axis (Fig. 2) through π–π interactions with a *Cg*1···*Cg*2^iv, v^ distance of 3.9517 (8) Å [symmetry codes: (iv) x, -1+y, z; (v) x, 1+y, z]. Weak C—H···π interactions (Table 1) are also present.

### Experimental

5-Ethyl-6-(3,5-dimethylbenzyl)uracil (0.258 g, 1 mmol) was stirred in anhydrous CH_3_CN (15 ml) under nitrogen and *N*,*O*-bis(trimethylsilyl)acetamide (0.87 ml, 3.5 mol) was added. After a clear solution was obtained (10 min), the reaction mixture was cooled to 223 K and trimethylsilyl trifluoromethanesulphonate (0.18 ml, 1 mmol) was added followed by dropwise addition of bis-(2-phenylethyloxy)methane (0.512 g, 2 mmol). The mixture was stirred at room temperature for 4 hr. The reaction was quenched with saturated aqueous NaHCO_3_ solution (5 ml) and evaporated under reduced pressure. The residue was extracted with ether (3 × 50 ml), the combined organic fractions were collected, dried (MgSO_4_) and evaporated under reduced pressure. The residue was chromatographed on silica gel column with CHCl_3_ to afford a white solid, which was recrystallized from ethanol to obtain the title compound as colorless crystals. Yield: 201 mg (51 %), M.p.: 396–398 K.

### Refinement

The amide H atom was located in a difference map and refined isotropically. The remaining H atoms were placed in calculated positions with d(C—H) = 0.93 for aromatic, 0.97 for CH_2_ and 0.96 Å for CH_3_ atoms. The *U*_iso_(H) values were constrained to be 1.5*U*_eq_ of the carrier atom for methyl H atoms and 1.2*U*_eq_ for the remaining H atoms. A rotating group model was used for the methyl groups.

### Crystal data

**Table:**

C_24_H_28_N_2_O_3_	*Z* = 2
*M**_r_* = 392.48	*F*(000) = 420
Triclinic, *P*1	*D*_x_ = 1.227 Mg m^−^^3^
Hall symbol: -P 1	Melting point = 396–398 K
*a* = 8.34310 (1) Å	Cu *K*α radiation, λ = 1.54178 Å
*b* = 8.47760 (1) Å	Cell parameters from 3863 reflections
*c* = 15.9821 (2) Å	θ = 2.8–70.0°
α = 91.5660 (1)°	µ = 0.65 mm^−^^1^
β = 91.7820 (1)°	*T* = 296 K
γ = 109.8350 (1)°	Block, colorless
*V* = 1061.97 (1) Å^3^	0.58 × 0.40 × 0.31 mm

### Data collection

**Table:**

Bruker SMART APEXII CCD area-detector diffractometer	3863 independent reflections
Radiation source: sealed tube	3542 reflections with *I* > 2σ(*I*)
Graphite monochromator	*R*_int_ = 0.022
φ and ω scans	θ_max_ = 70.0°, θ_min_ = 2.8°
Absorption correction: multi-scan (*SADABS*; Bruker, 2009)	*h* = −10→10
*T*_min_ = 0.705, *T*_max_ = 0.824	*k* = −10→10
13575 measured reflections	*l* = −19→19

### Refinement

**Table:**

Refinement on *F*^2^	Secondary atom site location: difference Fourier map
Least-squares matrix: full	Hydrogen site location: inferred from neighbouring sites
*R*[*F*^2^ > 2σ(*F*^2^)] = 0.044	H atoms treated by a mixture of independent and constrained refinement
*wR*(*F*^2^) = 0.123	*w* = 1/[σ^2^(*F*_o_^2^) + (0.0599*P*)^2^ + 0.2589*P*] where *P* = (*F*_o_^2^ + 2*F*_c_^2^)/3
*S* = 1.05	(Δ/σ)_max_ = 0.001
3863 reflections	Δρ_max_ = 0.27 e Å^−^^3^
270 parameters	Δρ_min_ = −0.18 e Å^−^^3^
0 restraints	Extinction correction: *SHELXTL* (Sheldrick, 2008), Fc^*^=kFc[1+0.001xFc^2^λ^3^/sin(2θ)]^-1/4^
Primary atom site location: structure-invariant direct methods	Extinction coefficient: 0.0158 (11)

### Special details

**Table:**

Geometry. All esds (except the esd in the dihedral angle between two l.s. planes) are estimated using the full covariance matrix. The cell esds are taken into account individually in the estimation of esds in distances, angles and torsion angles; correlations between esds in cell parameters are only used when they are defined by crystal symmetry. An approximate (isotropic) treatment of cell esds is used for estimating esds involving l.s. planes.
Refinement. Refinement of F^2^ against ALL reflections. The weighted R-factor wR and goodness of fit S are based on F^2^, conventional R-factors R are based on F, with F set to zero for negative F^2^. The threshold expression of F^2^ > 2sigma(F^2^) is used only for calculating R-factors(gt) etc. and is not relevant to the choice of reflections for refinement. R-factors based on F^2^ are statistically about twice as large as those based on F, and R- factors based on ALL data will be even larger.

### Fractional atomic coordinates and isotropic or equivalent isotropic displacement parameters (Å^2^)

**Table:**

	*x*	*y*	*z*	*U*_iso_*/*U*_eq_	
N1	0.73374 (14)	0.17782 (14)	0.18049 (7)	0.0389 (3)	
N2	0.86631 (15)	0.35816 (15)	0.07678 (7)	0.0416 (3)	
H1N2	0.855 (2)	0.413 (2)	0.0316 (12)	0.050 (4)*	
O1	1.15299 (13)	0.46537 (13)	0.07074 (6)	0.0516 (3)	
O2	0.57921 (14)	0.25880 (15)	0.08011 (7)	0.0586 (3)	
O3	0.56324 (12)	−0.10907 (12)	0.19600 (6)	0.0465 (3)	
C1	0.89379 (17)	0.18893 (16)	0.21512 (8)	0.0372 (3)	
C2	1.03943 (17)	0.28209 (17)	0.18009 (8)	0.0398 (3)	
C3	1.02860 (18)	0.37579 (17)	0.10647 (8)	0.0400 (3)	
C4	0.71675 (18)	0.26506 (17)	0.11021 (8)	0.0408 (3)	
C5	0.57628 (17)	0.05835 (18)	0.21083 (9)	0.0439 (3)	
H5A	0.5718	0.0791	0.2706	0.053*	
H5B	0.4793	0.0771	0.1835	0.053*	
C6	0.5432 (2)	−0.1640 (2)	0.10923 (9)	0.0496 (4)	
H6A	0.6137	−0.0757	0.0755	0.060*	
H6B	0.4253	−0.1918	0.0897	0.060*	
C7	0.5968 (2)	−0.3174 (2)	0.10163 (11)	0.0545 (4)	
H7A	0.5223	−0.4062	0.1337	0.065*	
H7B	0.5846	−0.3567	0.0434	0.065*	
C8	0.77867 (19)	−0.27984 (17)	0.13271 (9)	0.0457 (3)	
C9	0.8179 (2)	−0.33678 (19)	0.20831 (10)	0.0511 (4)	
H9A	0.7302	−0.4020	0.2402	0.061*	
C10	0.9850 (2)	−0.2985 (2)	0.23730 (11)	0.0561 (4)	
H10A	1.0087	−0.3380	0.2883	0.067*	
C11	1.1160 (2)	−0.2024 (2)	0.19109 (12)	0.0579 (4)	
H11A	1.2285	−0.1770	0.2105	0.070*	
C12	1.0798 (2)	−0.1441 (2)	0.11607 (12)	0.0614 (4)	
H12A	1.1682	−0.0782	0.0847	0.074*	
C13	0.9130 (2)	−0.1827 (2)	0.08704 (10)	0.0545 (4)	
H13A	0.8901	−0.1430	0.0360	0.065*	
C14	0.89232 (19)	0.09042 (18)	0.29230 (8)	0.0430 (3)	
H14A	1.0062	0.0871	0.3029	0.052*	
H14B	0.8170	−0.0241	0.2806	0.052*	
C15	0.83675 (18)	0.15601 (18)	0.37173 (8)	0.0433 (3)	
C16	0.8249 (2)	0.31421 (19)	0.38003 (9)	0.0491 (4)	
H16A	0.8513	0.3851	0.3353	0.059*	
C17	0.7738 (2)	0.3697 (2)	0.45442 (10)	0.0573 (4)	
C18	0.7354 (2)	0.2618 (3)	0.52015 (10)	0.0642 (5)	
H18A	0.7011	0.2972	0.5699	0.077*	
C19	0.7467 (2)	0.1027 (2)	0.51394 (9)	0.0597 (4)	
C20	0.7976 (2)	0.0516 (2)	0.43944 (9)	0.0523 (4)	
H20A	0.8059	−0.0549	0.4344	0.063*	
C21	0.7013 (3)	−0.0151 (3)	0.58581 (12)	0.0859 (7)	
H21A	0.7585	−0.0957	0.5808	0.129*	
H21B	0.5802	−0.0724	0.5842	0.129*	
H21C	0.7364	0.0481	0.6380	0.129*	
C22	0.7617 (3)	0.5431 (3)	0.46177 (14)	0.0814 (6)	
H22A	0.6780	0.5439	0.5015	0.122*	
H22B	0.7287	0.5734	0.4081	0.122*	
H22C	0.8705	0.6222	0.4803	0.122*	
C23	1.21678 (19)	0.2957 (2)	0.21143 (10)	0.0509 (4)	
H23A	1.2136	0.1883	0.2322	0.061*	
H23B	1.2922	0.3205	0.1649	0.061*	
C24	1.2888 (3)	0.4295 (3)	0.28019 (13)	0.0758 (5)	
H24A	1.3994	0.4298	0.2989	0.114*	
H24B	1.2142	0.4065	0.3263	0.114*	
H24C	1.2987	0.5372	0.2591	0.114*	

### Atomic displacement parameters (Å^2^)

**Table:**

	*U*^11^	*U*^22^	*U*^33^	*U*^12^	*U*^13^	*U*^23^
N1	0.0382 (6)	0.0428 (6)	0.0351 (5)	0.0130 (5)	0.0022 (4)	0.0040 (4)
N2	0.0461 (6)	0.0448 (6)	0.0340 (6)	0.0152 (5)	0.0002 (5)	0.0083 (5)
O1	0.0464 (6)	0.0569 (6)	0.0462 (6)	0.0096 (5)	0.0055 (4)	0.0146 (5)
O2	0.0461 (6)	0.0739 (8)	0.0567 (7)	0.0212 (5)	−0.0057 (5)	0.0165 (5)
O3	0.0450 (5)	0.0460 (5)	0.0447 (5)	0.0105 (4)	0.0018 (4)	0.0049 (4)
C1	0.0413 (7)	0.0390 (7)	0.0321 (6)	0.0148 (6)	−0.0005 (5)	−0.0003 (5)
C2	0.0419 (7)	0.0420 (7)	0.0349 (6)	0.0133 (6)	0.0006 (5)	0.0021 (5)
C3	0.0442 (7)	0.0393 (7)	0.0346 (6)	0.0120 (6)	0.0025 (5)	0.0006 (5)
C4	0.0435 (7)	0.0438 (7)	0.0364 (6)	0.0165 (6)	−0.0012 (5)	0.0019 (5)
C5	0.0387 (7)	0.0503 (8)	0.0418 (7)	0.0135 (6)	0.0060 (5)	0.0033 (6)
C6	0.0455 (8)	0.0550 (9)	0.0478 (8)	0.0177 (7)	−0.0073 (6)	−0.0035 (6)
C7	0.0501 (9)	0.0463 (8)	0.0630 (9)	0.0129 (7)	−0.0099 (7)	−0.0063 (7)
C8	0.0472 (8)	0.0377 (7)	0.0527 (8)	0.0161 (6)	−0.0016 (6)	−0.0064 (6)
C9	0.0487 (8)	0.0435 (8)	0.0588 (9)	0.0122 (7)	0.0035 (7)	0.0046 (6)
C10	0.0566 (9)	0.0526 (9)	0.0604 (9)	0.0208 (8)	−0.0064 (7)	0.0038 (7)
C11	0.0437 (8)	0.0571 (9)	0.0740 (11)	0.0190 (7)	−0.0016 (7)	−0.0018 (8)
C12	0.0506 (9)	0.0626 (10)	0.0719 (11)	0.0190 (8)	0.0167 (8)	0.0082 (8)
C13	0.0589 (9)	0.0562 (9)	0.0520 (9)	0.0240 (8)	0.0054 (7)	0.0045 (7)
C14	0.0465 (7)	0.0439 (7)	0.0388 (7)	0.0153 (6)	0.0008 (5)	0.0076 (5)
C15	0.0442 (7)	0.0470 (8)	0.0339 (7)	0.0093 (6)	−0.0027 (5)	0.0054 (5)
C16	0.0574 (9)	0.0488 (8)	0.0370 (7)	0.0127 (7)	0.0015 (6)	0.0039 (6)
C17	0.0604 (10)	0.0636 (10)	0.0446 (8)	0.0177 (8)	−0.0007 (7)	−0.0067 (7)
C18	0.0635 (10)	0.0863 (13)	0.0354 (8)	0.0165 (9)	0.0033 (7)	−0.0063 (7)
C19	0.0591 (9)	0.0743 (11)	0.0346 (7)	0.0082 (8)	−0.0019 (6)	0.0100 (7)
C20	0.0575 (9)	0.0543 (9)	0.0394 (7)	0.0113 (7)	−0.0019 (6)	0.0115 (6)
C21	0.0950 (15)	0.1073 (17)	0.0425 (9)	0.0158 (13)	0.0066 (9)	0.0258 (10)
C22	0.1037 (16)	0.0772 (13)	0.0676 (12)	0.0374 (12)	0.0066 (11)	−0.0150 (10)
C23	0.0451 (8)	0.0563 (9)	0.0510 (8)	0.0163 (7)	0.0024 (6)	0.0109 (7)
C24	0.0590 (11)	0.0903 (14)	0.0677 (11)	0.0142 (10)	−0.0150 (9)	−0.0076 (10)

### Geometric parameters (Å, º)

**Table:**

N1—C4	1.3913 (17)	C12—C13	1.379 (2)
N1—C1	1.4014 (17)	C12—H12A	0.9300
N1—C5	1.4664 (17)	C13—H13A	0.9300
N2—C4	1.3643 (18)	C14—C15	1.519 (2)
N2—C3	1.3783 (18)	C14—H14A	0.9700
N2—H1N2	0.887 (19)	C14—H14B	0.9700
O1—C3	1.2270 (17)	C15—C16	1.381 (2)
O2—C4	1.2141 (17)	C15—C20	1.3926 (19)
O3—C5	1.3992 (17)	C16—C17	1.398 (2)
O3—C6	1.4372 (18)	C16—H16A	0.9300
C1—C2	1.3524 (19)	C17—C18	1.385 (3)
C1—C14	1.5065 (17)	C17—C22	1.509 (3)
C2—C3	1.4548 (18)	C18—C19	1.385 (3)
C2—C23	1.5128 (19)	C18—H18A	0.9300
C5—H5A	0.9700	C19—C20	1.383 (2)
C5—H5B	0.9700	C19—C21	1.514 (2)
C6—C7	1.514 (2)	C20—H20A	0.9300
C6—H6A	0.9700	C21—H21A	0.9600
C6—H6B	0.9700	C21—H21B	0.9600
C7—C8	1.505 (2)	C21—H21C	0.9600
C7—H7A	0.9700	C22—H22A	0.9600
C7—H7B	0.9700	C22—H22B	0.9600
C8—C9	1.384 (2)	C22—H22C	0.9600
C8—C13	1.387 (2)	C23—C24	1.511 (3)
C9—C10	1.381 (2)	C23—H23A	0.9700
C9—H9A	0.9300	C23—H23B	0.9700
C10—C11	1.372 (2)	C24—H24A	0.9600
C10—H10A	0.9300	C24—H24B	0.9600
C11—C12	1.373 (3)	C24—H24C	0.9600
C11—H11A	0.9300		
			
C4—N1—C1	121.92 (11)	C12—C13—C8	121.12 (15)
C4—N1—C5	116.53 (11)	C12—C13—H13A	119.4
C1—N1—C5	121.18 (11)	C8—C13—H13A	119.4
C4—N2—C3	126.71 (11)	C1—C14—C15	116.09 (11)
C4—N2—H1N2	115.1 (11)	C1—C14—H14A	108.3
C3—N2—H1N2	118.2 (11)	C15—C14—H14A	108.3
C5—O3—C6	114.94 (11)	C1—C14—H14B	108.3
C2—C1—N1	121.22 (12)	C15—C14—H14B	108.3
C2—C1—C14	122.80 (12)	H14A—C14—H14B	107.4
N1—C1—C14	115.98 (11)	C16—C15—C20	118.73 (14)
C1—C2—C3	118.98 (12)	C16—C15—C14	123.13 (12)
C1—C2—C23	124.62 (12)	C20—C15—C14	118.14 (13)
C3—C2—C23	116.39 (12)	C15—C16—C17	121.23 (14)
O1—C3—N2	120.07 (12)	C15—C16—H16A	119.4
O1—C3—C2	123.99 (13)	C17—C16—H16A	119.4
N2—C3—C2	115.93 (12)	C18—C17—C16	118.24 (16)
O2—C4—N2	122.00 (12)	C18—C17—C22	121.61 (16)
O2—C4—N1	122.80 (13)	C16—C17—C22	120.15 (16)
N2—C4—N1	115.20 (11)	C17—C18—C19	121.94 (15)
O3—C5—N1	112.94 (11)	C17—C18—H18A	119.0
O3—C5—H5A	109.0	C19—C18—H18A	119.0
N1—C5—H5A	109.0	C20—C19—C18	118.37 (15)
O3—C5—H5B	109.0	C20—C19—C21	120.28 (18)
N1—C5—H5B	109.0	C18—C19—C21	121.34 (17)
H5A—C5—H5B	107.8	C19—C20—C15	121.50 (16)
O3—C6—C7	107.77 (12)	C19—C20—H20A	119.3
O3—C6—H6A	110.2	C15—C20—H20A	119.3
C7—C6—H6A	110.2	C19—C21—H21A	109.5
O3—C6—H6B	110.2	C19—C21—H21B	109.5
C7—C6—H6B	110.2	H21A—C21—H21B	109.5
H6A—C6—H6B	108.5	C19—C21—H21C	109.5
C8—C7—C6	111.71 (12)	H21A—C21—H21C	109.5
C8—C7—H7A	109.3	H21B—C21—H21C	109.5
C6—C7—H7A	109.3	C17—C22—H22A	109.5
C8—C7—H7B	109.3	C17—C22—H22B	109.5
C6—C7—H7B	109.3	H22A—C22—H22B	109.5
H7A—C7—H7B	107.9	C17—C22—H22C	109.5
C9—C8—C13	117.69 (14)	H22A—C22—H22C	109.5
C9—C8—C7	121.57 (14)	H22B—C22—H22C	109.5
C13—C8—C7	120.71 (14)	C24—C23—C2	113.15 (14)
C10—C9—C8	121.20 (15)	C24—C23—H23A	108.9
C10—C9—H9A	119.4	C2—C23—H23A	108.9
C8—C9—H9A	119.4	C24—C23—H23B	108.9
C11—C10—C9	120.18 (15)	C2—C23—H23B	108.9
C11—C10—H10A	119.9	H23A—C23—H23B	107.8
C9—C10—H10A	119.9	C23—C24—H24A	109.5
C10—C11—C12	119.56 (15)	C23—C24—H24B	109.5
C10—C11—H11A	120.2	H24A—C24—H24B	109.5
C12—C11—H11A	120.2	C23—C24—H24C	109.5
C11—C12—C13	120.24 (16)	H24A—C24—H24C	109.5
C11—C12—H12A	119.9	H24B—C24—H24C	109.5
C13—C12—H12A	119.9		
			
C4—N1—C1—C2	−1.78 (19)	C13—C8—C9—C10	−0.1 (2)
C5—N1—C1—C2	170.99 (12)	C7—C8—C9—C10	−178.47 (14)
C4—N1—C1—C14	178.71 (12)	C8—C9—C10—C11	0.0 (2)
C5—N1—C1—C14	−8.52 (17)	C9—C10—C11—C12	0.3 (3)
N1—C1—C2—C3	1.72 (19)	C10—C11—C12—C13	−0.4 (3)
C14—C1—C2—C3	−178.80 (12)	C11—C12—C13—C8	0.4 (3)
N1—C1—C2—C23	−177.60 (12)	C9—C8—C13—C12	−0.1 (2)
C14—C1—C2—C23	1.9 (2)	C7—C8—C13—C12	178.30 (14)
C4—N2—C3—O1	−179.20 (13)	C2—C1—C14—C15	109.79 (15)
C4—N2—C3—C2	1.31 (19)	N1—C1—C14—C15	−70.71 (16)
C1—C2—C3—O1	179.10 (13)	C1—C14—C15—C16	−14.3 (2)
C23—C2—C3—O1	−1.5 (2)	C1—C14—C15—C20	166.30 (13)
C1—C2—C3—N2	−1.44 (18)	C20—C15—C16—C17	−0.3 (2)
C23—C2—C3—N2	177.93 (12)	C14—C15—C16—C17	−179.73 (14)
C3—N2—C4—O2	179.05 (13)	C15—C16—C17—C18	0.1 (3)
C3—N2—C4—N1	−1.3 (2)	C15—C16—C17—C22	−179.92 (16)
C1—N1—C4—O2	−178.91 (13)	C16—C17—C18—C19	0.2 (3)
C5—N1—C4—O2	8.0 (2)	C22—C17—C18—C19	−179.82 (18)
C1—N1—C4—N2	1.45 (18)	C17—C18—C19—C20	−0.2 (3)
C5—N1—C4—N2	−171.63 (11)	C17—C18—C19—C21	−178.89 (18)
C6—O3—C5—N1	−67.31 (14)	C18—C19—C20—C15	−0.1 (3)
C4—N1—C5—O3	108.67 (13)	C21—C19—C20—C15	178.66 (16)
C1—N1—C5—O3	−64.47 (16)	C16—C15—C20—C19	0.3 (2)
C5—O3—C6—C7	159.11 (12)	C14—C15—C20—C19	179.75 (14)
O3—C6—C7—C8	−58.84 (17)	C1—C2—C23—C24	−85.87 (19)
C6—C7—C8—C9	106.27 (16)	C3—C2—C23—C24	94.80 (17)
C6—C7—C8—C13	−72.06 (19)		

### Hydrogen-bond geometry (Å, º)

*Cg*3 is the centroid of the C15–C20 ring.

**Table:**

*D*—H···*A*	*D*—H	H···*A*	*D*···*A*	*D*—H···*A*
N2—H1*N*2···O1^i^	0.889 (18)	1.969 (18)	2.8558 (15)	175.7 (17)
C14—H14*B*···O3	0.97	2.36	3.0349 (18)	126
C21—H21*B*···*Cg*3^ii^	0.96	2.90	3.845 (3)	170
C22—H22*C*···*Cg*3^iii^	0.96	2.92	3.861 (3)	166

Symmetry codes: (i) −*x*+2, −*y*+1, −*z*; (ii) −*x*+1, −*y*, −*z*+1; (iii) −*x*+2, −*y*+1, −*z*+1.

## References

[bb1] Allen, F. H., Kennard, O., Watson, D. G., Brammer, L., Orpen, A. G. & Taylor, R. (1987). *J. Chem. Soc. Perkin Trans. 2*, pp. S1–19.

[bb2] Bernstein, J., Davis, R. E., Shimoni, L. & Chang, N.-L. (1995). *Angew. Chem. Int. Ed. Engl* **34**, 1555–1573.

[bb3] Bruker (2009). *APEX2*, *SAINT* and *SADABS* Bruker AXS Inc., Madison, Wisconsin, USA.

[bb4] El-Brollosy, N. R., Al-Deeb, O. A., El-Emam, A. A., Pedersen, E. B., La Colla, P., Collu, G., Sanna, G. & Loddo, R. (2009). *Arch. Pharm. Chem. Life Sci* **342**, 663–670. 10.1002/ardp.200900139 19856332

[bb5] El-Brollosy, N. R., Al-Omar, M. A., Al-Deeb, O. A., El-Emam, A. A. & Nielsen, C. (2007). *J. Chem. Res* pp. 263–267.

[bb6] El-Brollosy, N. R., El-Emam, A. A., Al-Deeb, O. A. & Ng, S. W. (2011). *Acta Cryst.* E**67**, o2839. 10.1107/S1600536811039821 PMC3247578 22219883

[bb7] El-Brollosy, N. R., El-Emam, A. A., Al-Deeb, O. A. & Ng, S. W. (2012). *Acta Cryst.* E**68**, o316. 10.1107/S1600536811055723 PMC3275006 22346951

[bb8] El-Brollosy, N. R., Sorensen, E. R., Pedersen, E. B., Sanna, G., La Colla, P. & Loddo, R. (2008). *Arch. Pharm. Chem. Life Sci* **341**, 9–19. 10.1002/ardp.200700113 18161905

[bb9] Hopkins, A. L., Ren, J., Esnouf, R. M., Willcox, B. E., Jones, E. Y., Ross, C., Miyasaka, T., Walker, R. T., Tanaka, H., Stammers, D. K. & Stuart, D. I. (1996). *J. Med. Chem* **39**, 1589–1600. 10.1021/jm960056x 8648598

[bb10] Hopkins, A. L., Ren, J., Tanaka, H., Baba, M., Okamato, M., Stuart, D. I. & Stammers, D. K. (1999). *J. Med. Chem* **42**, 4500–4505. 10.1021/jm990192c 10579814

[bb11] Sheldrick, G. M. (2008). *Acta Cryst.* A**64**, 112–122. 10.1107/S0108767307043930 18156677

[bb12] Spek, A. L. (2009). *Acta Cryst.* D**65**, 148–155. 10.1107/S090744490804362X PMC2631630 19171970

